# Evaluation of the Insect Resistance Efficacy of Transgenic Maize LD05 in China

**DOI:** 10.3390/plants14193051

**Published:** 2025-10-02

**Authors:** Wenlan Li, Xinwei Hou, Hua Zhang, Xiaoyan Yang, Zhaohua Ding, Runqing Yue

**Affiliations:** 1 Shandong Key Laboratory of Maize Biological Breeding, Maize Research Institute, National Engineering Center of Wheat and Maize, Shandong Academy of Agricultural Sciences, Jinan 250100, China; liwenlantutu@126.com (W.L.); houxinwei92@163.com (X.H.); zhuanghua1986yms@163.com (H.Z.); 2 Institute of Biotechnology, Chongqing Academy of Agricultural Sciences, Chongqing 401329, China; yangxiaoyan89cq@163.com

**Keywords:** transgenic maize LD05, insect resistance, efficacy, China

## Abstract

Transgenic insect-resistant maize can effectively control insect pests, which is of great significance to improve maize yield and quality. Transgenic maize LD05 is an insect-resistant and herbicide-tolerant maize independently developed by Shandong Academy of Agricultural Sciences and highly resistant to major lepidopteran pests. In order to study the pest resistance of transgenic maize LD05 in different ecological areas of China, this study conducted a laboratory bioassay, and artificial inoculation test and natural pest investigation in field were carried out in one pilot of each of five maize ecological zones in China. The results of laboratory bioassay showed that transgenic maize LD05 had high resistance to *Ostrinia furnacalis* (Guenée), *Mythimna separata* (Walker), *Helicoverpa armigera* (Hübner) and *Spodoptera frugiperda* (J. E. Smith), the main lepidopteran pests threatening maize production in China. The results of artificial inoculation test and natural pest investigation in field showed that transgenic maize LD05 had high resistance to major lepidopteran pests in different ecological areas of China, which was consistent with the pest resistance management strategy, and can provide important theoretical basis and technical support for the industrialization of transgenic maize LD05 in the future.

## 1. Introduction

Corn (*Zea mays* L.), as one of the three major food crops in the world, occupies a dominant position in China’s food production, and its yield and quality are directly related to national food security [1,2]. However, the pest problem seriously restricts the sustainable development of China’s corn industry. Major pests such as *O. furnacalis*, *H. armigera* and the invasive insect *S. frugiperda* not only directly damage the growth of maize but also aggravate the occurrence of secondary diseases such as ear rot of maize [3,4]. Corn production is reduced by 15 to 20% annually due to pest infestations in China [5]. Although the traditional chemical control methods have a remarkable effect in the short term, long-term use easily leads to insect resistance, reduces the control effect and brings about secondary problems such as environmental pollution [2,3]. The development and application of transgenic insect-resistant maize provide a more efficient and environmentally friendly solution for integrated pest management.

Since the first transgenic insect-resistant maize was commercially planted in 1996, remarkable progress has been made in the research and development of transgenic maize worldwide [6]. According to statistics, more than 244 transgenic maize conversion events have been approved, 210 of which contain insect resistance traits, fully reflecting the core position of insect resistance traits in the research and development of transgenic maize [2,3]. International seed giants such as Monsanto, DuPont Pioneer and Bayer have successfully developed a series of insect-resistant maize varieties and gene-superimposed varieties such as MON810, TC1507 and MIR162, effectively controlling the damage of lepidopteran pests such as *O. furnacalis* and *S. frugiperda*, significantly increasing economic, social and ecological benefits [2]. China has cultivated a number of transgenic maize transformants with independent intellectual property rights, and by 2024, 25 transgenic insect-resistant and herbicide-tolerant corn transformants have obtained production and application safety certificates issued by the Ministry of Agriculture and Rural Affairs transformants, which have demonstrated effective prevention and control capabilities against *O. furnacalis* and *S. frugiperda*, laying a solid foundation for industrial application [7,8,9].

A major threat to the sustainable cultivation of transgenic insect-resistant maize is the development of resistance to target pests. As insect-resistant transgenic maize has been widely planted in the United States for many years, field monitoring data show that there are transformed resistant to target pests. For example, populations of *Ostrinia nubilalis* (Hübner) have developed resistance to traditional Bt proteins such as Cry1Ab, Cry1F and Cry2A [10,11,12,13]. However, “high dose/refuge” and “pyramid” strategies have become effective means to delay the development of pest resistance [14,15]. The synergistic application of Cry protein with Vip3 protein, which has a different mechanism from traditional Cry protein, can not only significantly expand the insecticidal spectrum, but also reduce the risk of resistance through multi-target effects. International seed industry companies have generally adopted the gene stacking technology of Cry and Vip3Aa as the research and development direction of a new generation of insect-resistant maize [16]. Different from gene superposition mainly achieved through hybridization polymerization at home and abroad, the insect resistance gene *m2cryAb-vip3A* used in the transformation event of transgenic maize LD05 developed by China was obtained through the innovative use of the main structural fusion strategy of cry1Ab and vip3A(a) [17]. It is highly resistant to major lepidopteran pests such as *O. furnacalis*, *S. frugiperda*, *M. separata* and *H. armigera*.

China has a wide maize planting area, exceeding 40 million hectares in 2023. Due to its vast territory, China’s maize cultivation spans five distinct ecological zones, each with unique climatic, soil and pest characteristics, creating a complex environment for evaluating transgenic maize performance. *O. furnacalis* was the main insect pest in Northeast Spring corn area (NeSCR) [18,19]. The Huang-Huai-Hai summer maize area (HHHSCR) was mainly affected by *O. furnacalis*, *M. separata* and *H. armigera* [18,19]. The Southwest (SwHCR) and Southeast Hill Maize areas (SeHCR) are seriously threatened by *S. frugiperda* [18,19]. In the Northwest Inland Maize Region (NwICR) and the Qinghai–Tibet Plateau maize Region (QZPCR), the *O. furnacalis* was the main pest [18,19]. At present, the evaluation of insect resistance of transgenic maize LD05 in different ecological regions is still insufficient. In order to evaluate the environmental adaptability and insect resistance stability of transgenic maize LD05 in different ecological regions of China, this study selected one test site in different ecological regions (Harbin, Shihezi, Jinan, Chongqing and Guangzhou). Insect resistance of LD05 under different ecological conditions was systematically monitored through a combination of laboratory bioassay and field trials. The trials were conducted in compliance with the laws, regulations and regulatory requirements of the Department of Agriculture regarding genetically modified crops. The research results can not only provide a regional application scheme for the commercial planting of insect-resistant transgenic corn LD05 but also provide important technical support for the industrialization development of transgenic corn in China.

## 2. Materials and Methods

### 2.1. Bt Corn and Non-Bt Corn

Transgenic maize LD05 was an insect-resistant transformant containing *m2cryAb-vip3A* gene, Zheng 58 (female parent of the commercial maize hybrid Zheng 958) was its non-transgenic control, Xianda 205 was local insect-susceptible control of Harbin, Jinan and Chongqing, Xinyu 21 was local insect-susceptible control of Shihezi and Xianyu 335 was local insect-susceptible control of Guangzhou; all provided by Shandong Academy of Agricultural Sciences (Jinan, Shandong, China).

### 2.2. Bioassays Using Plant Tissues

The tested insects *O. furnacalis*, *M. separata*, *H. armigera* and *S. frugiperda* population were collected from Guangdong, China in 2020. The tested insects *O. furnacalis*, *M. separata*, *H. armigera* and *S. frugiperda* were all raised in the laboratory for more than 2 generations, with relatively consistent genetic background, and had not been exposed to any chemical pesticides or insect-resistant transgenic plant materials in the past 2 generations.

The transgenic corn LD05 and the control Z58 adopted a random block design, with three replicates. The block area was 30 m^2^ (5 m × 6 m, row spacing 60 cm, plant spacing 25 cm). The management is the same as that of conventional cultivation, and no pesticides were sprayed throughout the entire growth period. Twenty normally growing corn plants were randomly selected from each plot. Unopened young heart leaves were collected at the V5 stage, ear leaves were collected at the RT stage and the R1 stage, tassels (5 cm long closed tassels) were collected at the R1 stage, silk (5 cm long unpollinated young silks) and bracts were collected at the R2 stage, and kernels (young seeds of 15 to 20 days after pollination) were collected during the R3 period. The collected samples were quickly placed in insect-catching containers, and laboratory bioassay was conducted, respectively, with the newly hatched larvae of *O. furnacalis*, *M. separata*, *H. armigera* and *S. frugiperda*.

For leaves, bracts, tassels and silks: Twenty plants were randomly sampled, and their target tissues were placed in rearing containers; each tissue sample was infested with 5 neonate larvae (20 replicates total). For kernels: Five kernels were collected from each of 20 plants and placed in multi-well rearing boards (one kernel per well), with 1 larva infested per well (20 replicates total). After infestation, the larvae were reared in an insect chamber set at 26–28 °C, with a photoperiod of 16 h:8 h (L:D) and relative humidity of 70–80%. Fresh plant tissues were replenished daily according to larval consumption. Larval survival was recorded daily for 5 consecutive days; larvae were considered dead if they showed no response or significantly inhibited growth when prodded with a fine brush. The calculation formula for the mortality rate is x = (N − n)/N × 100%, where x represents the mortality rate, n represents the number of survivors and N represents the number of infections. The calculation formula for the corrected mortality rate is y = (x_t_ − x_0_)/(1 − x_0_) × 100%, where y represents the corrected mortality rate, x_t_ represents the mortality rate of the test material and x_0_ represents the mortality rate of the insect-infected control material. The criteria for judging insect resistance based on the corrected mortality rate are detailed in Table 1.

### 2.3. Field Trials

Field trials were conducted in Harbin (Minzhu Town, Daowai District, Harbin City, Heilongjiang Province), Shihezi (Agricultural park, Beiquan Town, Shihezi City, Xinjiang Uygur Autonomous Region), Jinan (Dangjia Town, Zhangqiu District, Jinan City, Shandong Province), Chongqing (Baishi Yi Town, Jiulongpo District, Chongqing) and Guangzhou (Ningxi Town, Zengcheng District, Guangzhou City, Guangdong Province), representing the five major ecological zones of corn in China, respectively. The test sites in Harbin, Shihezi, Jinan, Chongqing and Guangzhou were sown on 20 May, 25 May, 15 May, 5 April and 15 April 2024, respectively, and the harvest was carried out 120 days later.

A randomized block design was used in the field artificial inoculation experiment, and each treatment was set up with three replicates. The area of each treatment plot was 30 m^2^ (5 m × 6 m, row spacing 60 cm, plant spacing 25 cm). The management was the same as that of conventional cultivation, and no insecticides were sprayed throughout the entire growth period. An interval of 2 m between different pest inoculation test plots was used to avoid the spread of pests between different plots. Insects were received on the heart leaf cluster of corn at the V5 stage or in the silk cluster at the R1 stage, respectively. Each period should be infested twice with a 3-day interval. For each treatment in each period, no less than 40 plants should be manually infested. Each plant should be infested with 30 to 40 newly hatched larvae of *O. furnacalis* (or 30 to 40 newly hatched larvae of *M. separata*, or 20 to 30 *H. armigera*, or 20 to 30 *S. frugiperda*). After 14 to 21 days of insect exposure during the V5 period, the feeding situation of the upper and middle leaves of the corn plants by the target pests was investigated one by one. The leaf feeding levels were determined according to Table 2 [20], and then the resistance levels of the materials to the target pests were classified and identified based on the average values of the leaf feeding levels (Table 3) [20]. After 14 to 21 days of insect exposure during the R1 period, the damage status of female corn ears, number of holes, tunnel length and number of live insects were investigated plant by plant. The damage level of female ears was determined according to Table 4 [20], and then the resistance level of the identification materials to the target pests was divided based on the average value of the damage level of female ears (Table 5) [20].

### 2.4. Statistics and Analysis

The corrected mortality was analyzed using an analysis of variance (ANOVA) and Kruskal–Wallis nonparametric test with Duncan’s multiple comparisons. Data from field trials were analyzed with an ANOVA test with Duncan’s multiple comparisons and a two-sample t-test for significant differences. SPSS 20.0 software was used for statistical analysis of the test data.

## 3. Results

### 3.1. Bioassay Analysis of LD05 Tissue on Different Lepidopteran Larvae

**Figure 1:**
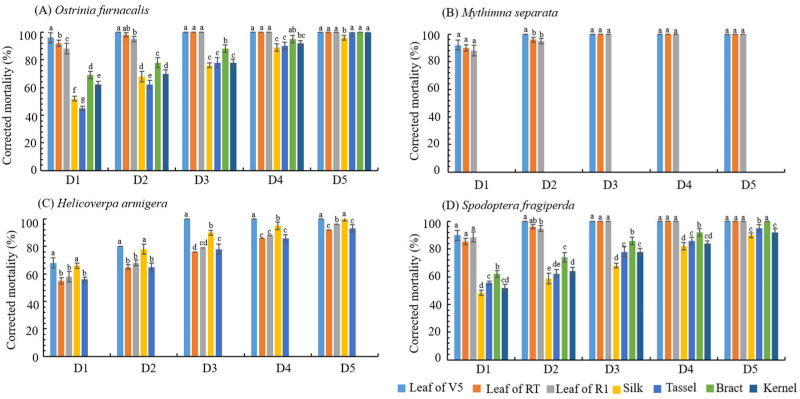
Corrected mortality of different lepidopteran neonate that fed on different tissues of LD05 corn for different durations. Error bars represent SE. Different lowercase letters combination above error bars for the same treatment time indicate a significant difference in mortality among tissues by Duncan’s multiple range test at the 0.05 level. D1: day 1; D2: day 2; D3: day 3; D4: day 4; D5: day 5.

In order to identify the resistance of transgenic maize LD05 to *O. furnacalis*, *M. separata*, *H. armigera* and *S. frugiperda*, according to the occurrence of different lepidopteran pests in maize growth period, tissues of different periods were selected to carry out laboratory bioassay on the target pests, and the results are shown in Figure 1. The ANOVA and Kruskal–Wallis test indicated that the corrected mortality of different lepidopteran larvae neonates feeding on different tissues of LD05 corn differed significantly among the treatment durations (Figure 1 and Table 6). The corrected mortality of *O. furnacalis* neonate of transgenic maize LD05 was 100.00% in all the seven tissues tested, except in the silk (only 96%). Among them, in the leaves of different periods, the corrected mortality can reach 100% in three days. The corrected mortality of *M. separata* neonate in transgenic maize LD05 was 100.00% at 3 days in leaves of different periods. The corrected mortality of *H. armigera* neonate of transgenic maize LD05 reached 100.00% at 3 days in the leaves of R1 stage and 100.00% at 5 days in the bracts during R1 period, and exceeded 90% in silk, tassel and kernel, all of which reached high resistance level. The corrected mortality of *S. frugiperda* neonate of transgenic maize LD05 reached 100.00% in leaves (V5, RT and R1) and bracts at different periods, and exceeded 90% in other tissues, all of which reached high resistance level.

### 3.2. Insect Resistance Identification of Transgenic Maize LD05 in Harbin

**Figure 2:**
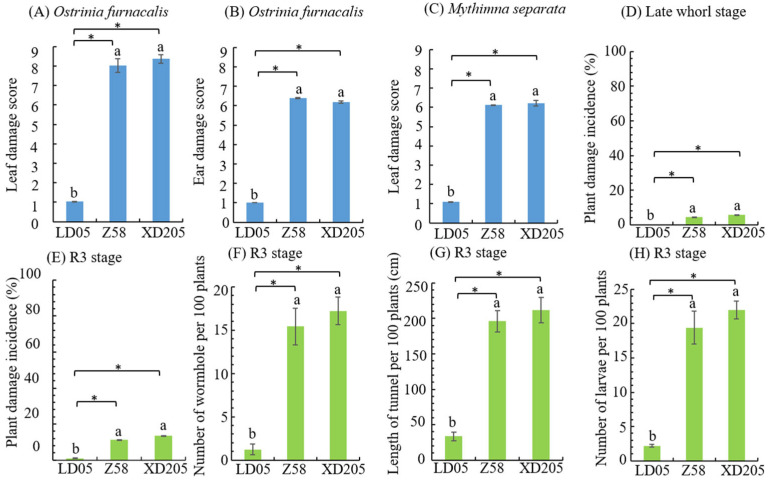
Investigation results of artificial inoculation and natural insect infestation in the field (Harbin). Z58 represents Zheng 58 and XD205 represents Xianda 205. Different lowercase letters above error bars indicate a significant difference in different treatments by Duncan’s multiple range test at the 0.05 level. Asterisks (*: *p* < 0.05) above the bar indicate a significant difference.

In Harbin, *O. furnacalis* and *M. separata* were the main insect pests in the field, and these two representative insects were selected in field artificial inoculation. In the field, *O. furnacalis* and *M. separata* were exposed at the heart leaf period, respectively. The leaf damage score of transgenic maize LD05 were 1.03 and 1.09, respectively, which were significantly lower than those of non-transgenic maize Zheng58 and insect-susceptible control maize Xianda 205, and the resistance level was high (Figure 2A,C). The results of inoculation of *O. furnacalis* in silking stage showed that the damage score of female ears of transgenic maize LD05 was 1.00 (Figure 2B), which was significantly lower than that of Zheng 58 and Xianda 205, and the resistance level was high.

In order to observe the natural occurrence of insect pests in the field, the statistical observation of lepidopteran pests in the experimental field was carried out. The major lepidopteran pests were *O. furnacalis* and *M. separata* in the heart leaf stage and *O. furnacalis* in the ear stage. No other lepidopteran pests were found. No lepidopteran pests were found at the early stage of heart leaf, and the plant damage rate of transgenic maize LD05 at the late stage of heart leaf was 0.99, while that of Zheng 58 and Xianda 205 were 11.29 and 13.38, respectively, with significant differences (Figure 2D). The plant damage incidence and results of panicle and pole dissection (number of holes, tunnel length and number of live insects) of transgenic corn LD05 at ear stage were significantly lower than those of non-transgenic corn Zheng 58 and insect-sensitive control Xanda 205 (Figure 2E–H).

### 3.3. Insect Resistance Identification of Transgenic Maize LD05 in Shihezi

**Figure 3:**
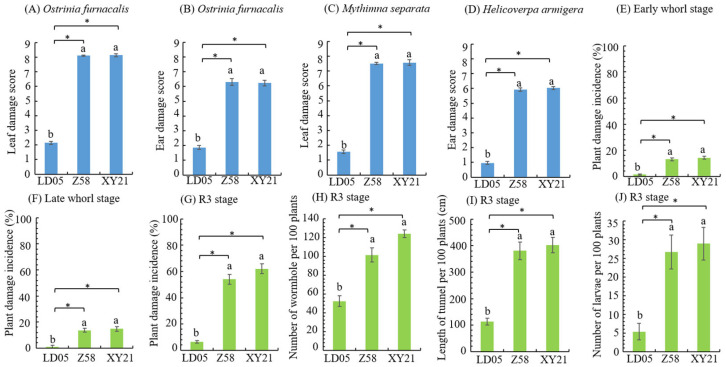
Investigation results of artificial inoculation and natural insect infestation in the field (Shihezi). Z58 represents Zheng 58 and XY21 represents Xinyu 21. Different lowercase letters above error bars indicate a significant difference in different treatments by Duncan’s multiple range test at the 0.05 level. Asterisks (*: *p* < 0.05) above the bar indicate a significant difference.

In Shihezi, because the main pests in the field were *O. furnacalis*, *M. separata* and *H. armigera*, these three representative insects were selected in the field artificial inoculation. In the field, *O. furnacalis* and *M. separata* were exposed at the heart leaf period, respectively. The leaf damage score of transgenic maize LD05 was significantly lower than that of non-transgenic maize Zheng58 and insect-susceptible control maize Xinyu 21, and the resistance level was high (Figure 3A,C). The results of field investigation of *O. furnacalis* and *H. armigera* in silking stage showed that the female ear damage score of transgenic maize LD05 was lower than Zheng 58 and Xinyu 21, and the difference was significant, and the resistance level was high (Figure 3B,D).

In order to observe the incidence of natural insect pests in the field, the statistical observation of lepidopteran pests in the experimental field was carried out. The main pests of lepidopteran were *O. furnacalis* and *M. separata* in the heart leaf stage, and *O. furnacalis* and *H. armigera* in the ear stage. The plant damage incidence of transgenic maize LD05 at the early and late heart leaf stages were 1.34 and 1.00, respectively, while the plant damage incidence of Zheng 58 and Xianyu 21 were both higher than 10%, with significant differences (Figure 3E,F). The plant damage incidence and results of panicle and pole dissection (number of holes, tunnel length and number of live insects) of transgenic corn LD05 at ear stage were significantly lower than Zheng 58 and Xianyu 21 (Figure 3G–J).

### 3.4. Insect Resistance Identification of Transgenic Maize LD05 in JINAN

**Figure 4:**
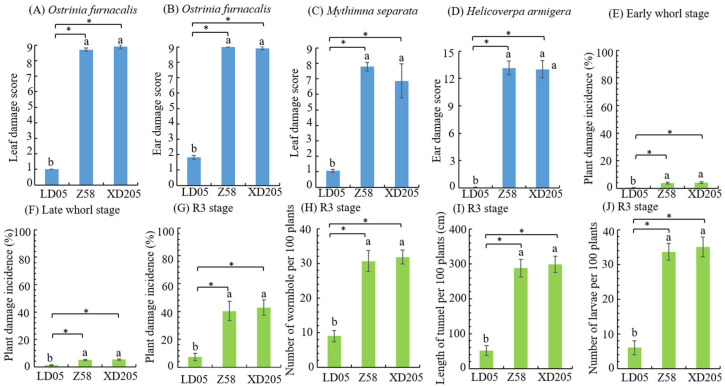
Investigation results of artificial inoculation and natural insect infestation in the field (Jinan). Z58 represents Zheng 58 and XD205 represents Xianda 205. Different lowercase letters above error bars indicate a significant difference in different treatments by Duncan’s multiple range test at the 0.05 level. Asterisks (*: *p* < 0.05) above the bar indicate a significant difference.

In Jinan, the major lepidopteran pests in the field were *O. furnacalis*, *M. separata* and *H. armigera*, which were selected by the field artificial inoculation. *O. furnacalis* and *M. separata* were exposed to the fields during heart leaf period, and the leaf damage score of transgenic maize LD05 were 1.00 and 1.06, respectively, which was significantly different from that of non-transgenic maize Zheng 58 and insect-susceptible control maize Xianda 205, and the resistance level was high (Figure 4A,C). *O. furnacalis* and *H. armigera* were exposed to the fields during the silking stage, and the female ear damage score of transgenic maize LD05 was significantly lower than Zheng 58 and Xianda 205, and the resistance level was high (Figure 4B,D).

In order to observe the incidence of natural insect pests in the field, the statistical observation of lepidopteran pests in the experimental field was carried out. The main pests of lepidopteran were *O. furnacalis* and *M. separata* in the heart leaf stage, and *O. furnacalis* and *H. armigera* in the ear stage. Plant damage incidence of transgenic maize LD05 at the early and late heart leaf stages were lower than Zheng 58 and Xianda 205, with significant differences (Figure 4E,F). The plant damage incidence and results of panicle and pole dissection (number of holes, tunnel length and number of live insects) of transgenic maize LD05 in ear stage were significantly lower than Zheng 58 and Xianda 205 (Figure 4G–J).

### 3.5. Insect Resistance Identification of Transgenic Maize LD05 in Chongqing

**Figure 5:**
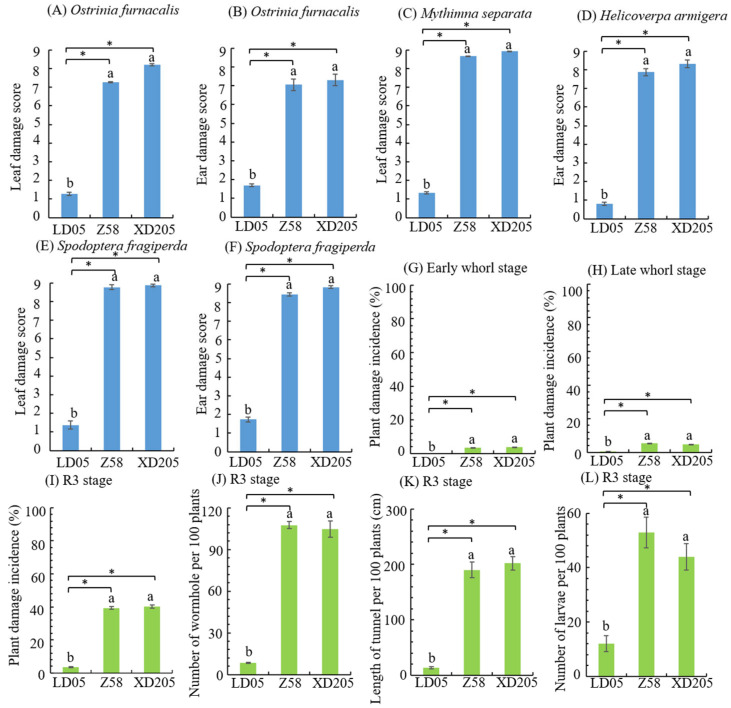
Investigation results of artificial inoculation and natural insect infestation in the field (Chongqing). Z58 represents Zheng 58 and XD205 represents Xianda 205. Different lowercase letters above error bars indicate a significant difference in different treatments by Duncan’s multiple range test at the 0.05 level. Asterisks (*: *p* < 0.05) above the bar indicate a significant difference.

In Chongqing, the main pests of lepidopteran in the field were *O. furnacalis*, *M. separata*, *H. armigera* and *S. frugiperda*. In the heart leaf stage, the *O. furnacalis*, *M. separata* and *S. frugiperda* were exposed to the fields, respectively. The investigation results showed that the leaf damage score of transgenic maize LD05 in heart leaf stage were significantly lower than those of non-transgenic maize Zheng 58 and insect-sensitive control maize Xianda 205, and the resistance levels were high (Figure 5A,C,E). *O. furnacalis*, *H. armigera* and *S. frugiperda* were exposed to the fields during the silking stage, and the investigation results showed that the female ear damage score of transgenic maize LD05 was significantly lower than Zheng 58 and Xianda 205, and the resistance level was high (Figure 5B,D,F).

In order to observe the incidence of natural insect pests in the field, the statistical observation of lepidopteran pests in the experimental field was carried out. The main pests of lepidopteran were *O. furnacalis*, *M. separata* and *S. frugiperda* in the heart leaf stage, and *O. furnacalis*, *H. armigera* and *S. frugiperda* in the ear stage. The plant damage incidence of transgenic maize LD05 at the early and late heart leaf stages was significantly lower than Zheng 58 and Xianda 205 (Figure 5G,H). The plant damage incidence and results of panicle and pole dissection (number of holes, tunnel length and number of live insects) of transgenic corn LD05 were significantly lower than Zheng 58 and Xianda205 (Figure 5I,J).

### 3.6. Insect Resistance Identification of Transgenic Maize LD05 in Guangzhou

**Figure 6:**
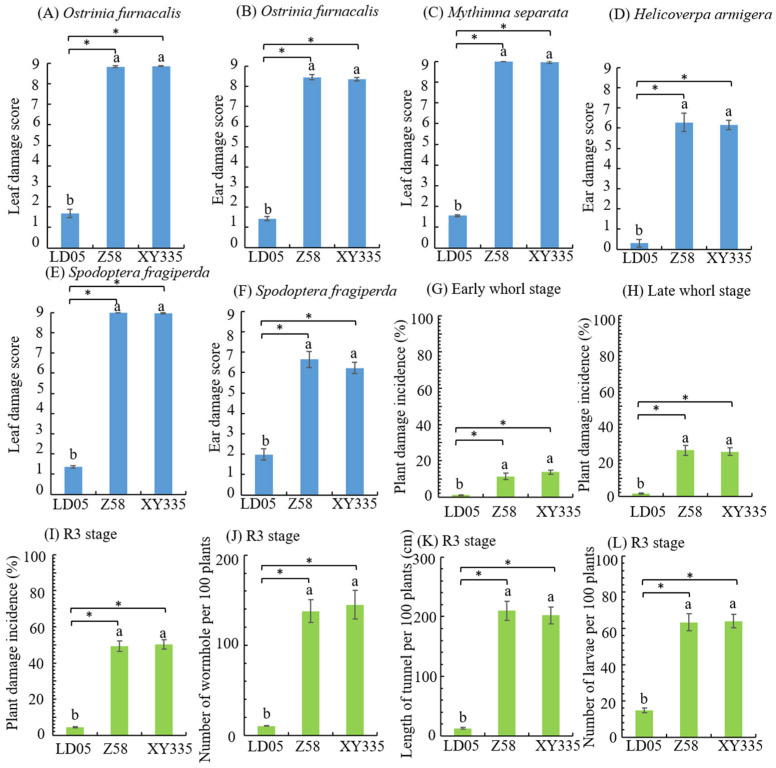
Investigation results of artificial inoculation and natural insect infestation in the field (Guangzhou). Z58 represents Zheng 58 and XY335 represents Xianyu 335. Different lowercase letters above error bars indicate a significant difference in different treatments by Duncan’s multiple range test at the 0.05 level. Asterisks (*: *p* < 0.05) above the bar indicate a significant difference.

In Guangzhou, the main pests of lepidopteran in the field were *O. furnacalis*, *M. separata*, *H. armigera* and *S. frugiperda*. These four representative insects were selected in the field artificial inoculation. In the heart leaf stage, the *O. furnacalis*, *M. separata* and *S. frugiperda* were exposed to the fields, respectively. The leaf damage score of transgenic corn LD05 in heart leaf stage was significantly lower than those of non-transgenic corn Zheng 58 and insect-sensitive control corn Xianyu 335, and the resistance levels were high (Figure 6A,C,E). The *O. furnacalis*, *H. armigera* and *S. frugiperda* were exposed to the fields during the silking stage, and the investigation results showed that the female ear damage score of transgenic maize LD05 was significantly lower than Zheng 58 and Xianyu 335, and the resistance level was high (Figure 6B,D,F).

In order to observe the incidence of natural insect pests in the field, the statistical observation of lepidopteran pests in the experimental field was carried out. The main pests of lepidopteran were *O. furnacalis*, *M. separata* and *S. frugiperda* in the heart leaf stage, and *O. furnacalis*, *H. armigera* and *S. frugiperda* in the ear stage. The plant damage incidence of transgenic maize LD05 at the early and late heart leaf stages was significantly lower than Zheng 58 and Xianyu 335 (Figure 6G,H). The plant damage incidence and results of panicle and pole dissection (number of holes, tunnel length and number of live insects) of transgenic corn LD05 were significantly lower than Zheng 58 and Xianyu 335 (Figure 6I,J).

### 3.7. Efficacy of Transgenic Maize LD05 Against Lepidopteran Pests in Different Experimental Sites

**Figure 7:**
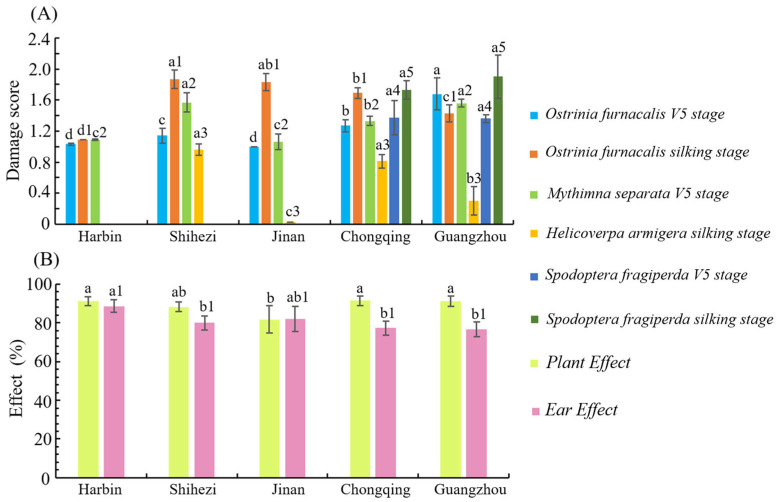
Efficacy of transgenic maize LD05 against lepidopteran pests in different experimental sites. (**A**): The damage score of lepidopteran pests to LD05 in V5 and silking stage at different sites. (**B**): Efficacy of LD05 plants and ears on lepidopteran pests at different sites. Different lowercase letters (or lowercase letters and numbers) above error bars indicate a significant difference in different treatments by Duncan’s multiple range test at the 0.05 level.

The insect resistance of transgenic maize LD05 in five different experimental sites was statistically analyzed. The results showed that after artificial inoculation of *O. furnacalis*, *M. separata* and *H. armigera* in the field, there were significant differences in the damage levels of LD05, but all reached the high resistance standard (Figure 7A). After artificial inoculation of *S. frugiperda* in the field, there was no significant difference in the damage level of LD05 between Chongqing and Guangzhou, and both reached high resistance level (Figure 7A). Under the natural occurrence of pests in the field, the efficacy of LD05 on lepidopteran pests in different test sites was calculated in the R3 period (Figure 7B). The statistical results showed that the pest control effect of LD05 maize plants in Harbin, Shihezi, Jinan, Chongqing and Guangzhou were 91.23%, 88.23%, 81.82%, 91.53% and 91.22%, respectively, with significant difference. The control effect of ear was slightly lower than that of plants (except Jinan), 88.66%, 80.01%, 82.18%, 77.36% and 76.66%, respectively, with significant differences (Figure 7B).

## 4. Discussion

The insect-resistant gene *m2cryAb-vip3A* used in it is a fusion gene with independent intellectual property rights that is obtained by designing and optimizing the main structural domains of the cry1Ab and vip3A(a) genes, which enables a single gene expression protein to have the activities of two types of insecticidal proteins. It shows high resistance to major corn pests such as *O. furnacalis*, *M. separata*, *H. armigera* and *S. frugiperda* throughout the entire growth period [17]. Compared with multi-gene polymerization materials, transgenic maize LD05 has a simpler program when introducing target traits into other receptor materials, which can effectively save backcross time and detection cost. In addition, the insect resistance gene expression box takes up less space in the vector, which can leave more space for efficient polymerization of disease-resistance and other genes, and realize the development of high-generation products in a short time.

The insecticidal effect of exogenous Bt in insect-resistant transgenic maize depends on its insecticidal protein expression [21,22]. Laboratory bioassay results showed that the corrected mortality of target pests in different tissues of transgenic maize LD05 was very different, suggesting that the concentration of Bt toxins is different in tissues and organs at different developmental stages, as found in DBN9936 (Cry1Ab), MON810 (Cry1Ab), MON88017(Cry3Bb1) and MIR162 (Vip3Aa20) [23,24,25,26]. In addition, laboratory bioassay results showed that the corrected mortality of transgenic maize LD05 leaves against target pests at different development stages could reach 100% on the third day, which was significantly higher than that of bracts, tassel, silk and kernels, which was consistent with previous research results [25,27]. The silk is a relatively weak expression site of the Bt protein [17], but the corrected mortality rate of the silk of the transgenic corn LD05 exceeded 90%, which could also reach a high resistance level. This is conducive to the implementation of the high-dose/refuge strategy by the country in resistance management.

The main maize producing areas in China were divided into six maize ecological zones according to geographical location. In the NeSCR, the spring is dry and windy, while the summer is warm and rainy, which is conducive to the growth and development of corn. In the HHHSCR, the early growth stage of corn is dry, and in the later stage, it is rainy with insufficient sunlight, resulting in serious diseases and pests. In the NwICR, with scarce rainfall, abundant sunlight and a large temperature difference between day and night, the quality of corn is excellent. In the SwHCR, the terrain is complex and the climatic conditions vary greatly. The climate in mountainous areas usually has a vertical distribution characteristic. As the altitude increases, the temperature drops and the precipitation increases. SeHCR climate is warm and humid, has abundant rainfall and is conducive to the growth of corn, but also easy to cause pests and diseases.

The main lepidopteran target pests in the NeSCR are *O. furnacalis* and *M. separata*; in the HHHSCR, the main pests are *O. furnacalis*, *M. separata* and *H. armigera*, etc.; in the NwICR, *O. furnacalis* is the main pest [18], and the transformants containing *cry* gene could effectively control the insect pests [25]. The *O. furnacalis* and the newly invaded *S. frugiperda* are the main pests in the SwHCR and SeHCR, the transformants containing only a single *cry* gene or a single *vip3* gene were difficult to control pests effectively [28]. Therefore, it has become a development trend to achieve multivalent insect resistance gene aggregation through gene reading frame tandem or “pyramid” strategy, such as transformants ND207, Zheda Ruifeng 8, DBN3601T, Bt11×MIR162×GA21, etc. [28], and these transgenic insect-resistant maize products showed good commercial prospects [24,25]. Based on the biological characteristics of the main pests in corn-growing areas, such as their distribution and host damage [19,29], we selected five experimental sites in different ecological zones to conduct field trials of LD05. The results of field artificial inoculation showed that transgenic maize LD05 could achieve high resistance to maize main pests such as *O. furnacalis*, *M. separata*, *H. armigera* and *S. frugiperda* in different ecological zones in China, which was consistent with previous research results [17]. The results of the natural occurrence of pests in the field indicated that the control efficacy of transgenic maize LD05 on major lepidopteran pests exhibited significant variation across different test sites yet consistently exceeded 80%. This indicates that LD05 can highly resist major lepidopteran pests such as *O. furnacalis* and *S. frugiperda* and is suitable for the main corn ecological zones in China. This is conducive to the implementation of China’s planting strategy for transgenic insect-resistant corn, which is based on the regional occurrence of major pests in corn-growing areas and the biological characteristics of host damage, and adopts a “zonal layout and source control” approach, as well as the high-dose/refuge resistance management strategy that suits China’s national conditions.

The main strategies for managing transgenic resistance include the “high dose/refuge” strategy and the “pyramid” strategy [14]. In the “high dose/refuge“ strategy, the high-dose expression of Bt insecticidal protein in Bt gene-converted crops is the key to the success of the implementation of the “high dose/refuge“ strategy. The expression level of *m2cryAb-vip3A* in different tissues of LD05, being the highest in the leaves at the seedling stage and the lowest in the mature roots. However, the results of laboratory bioassay and field experiments show that all tissues have achieved a high resistance level to the target pests, which provides an important scientific basis for the formulation and implementation of the “high dose/refuge“ strategy in the future. Individual insecticidal proteins often have a relatively narrow insecticidal spectrum and low insecticidal activity. Long-term and excessive use can lead to the development of pest resistance [30]. The protein expressed by *m2cryAb-vip3A* fusion insecticidal genes has both cry1Ab and vip3A(a) insecticidal activities, and these two proteins have different sources and insecticidal mechanisms. M2CryAb-Vip3A is a novel protein-based insecticidal toxin with high insecticidal activity, which has significant application value in enhancing insecticidal ability and slowing down the occurrence of pest resistance [16]. The laboratory bioassay and field trials in different ecological zones of GM maize LD05 not only provided scientific guarantee for the safe application of GM maize LD05 in China, but also met the regulatory requirements, significantly reduced the risk of variety promotion and would effectively promote the accurate layout and sustainable use of insect-resistant maize varieties.

## 5. Conclusions

In summary, the laboratory bioassay results showed that transgenic maize LD05 had high resistance to *O. furnacalis*, *M. separata*, *H. armigera* and *S. frugiperda*. The results of field artificial inoculation and natural occurrence of field pests in different ecological zones in China showed that transgenic maize LD05 had high resistance to major lepidopteran pests. The results will provide theoretical basis and technical support for insect resistance and industrialization of transgenic maize LD05.

## Figures and Tables

**Table 1:** Criteria for determining insect resistance.

Resistance Level	Larval Corrected Mortality Rate (y) %	
Test insects	*O. furnacalis*	*M. separata*/*H. armigera*/*S. frugiperda*
High resistance	y ≥ 95	y ≥ 90
Resistance	95 > y ≥ 60	90 > y ≥ 60
Medium resistance	60 > y ≥ 40	60 > y ≥ 40
Sense	40 > y	40 > y

**Table 2:** Grading criteria for the degree of damage to heart leaves caused by *O. furnacalis*, *M. separata* and *S. frugiperda.*

Leaf Feeding Level	Description of Symptoms of *O. furnacalis*	Description of Symptoms of *M. separata*	Description of Symptoms of *S. frugiperda*
1	Only a few individual leaves have 1–2 wormholes with a diameter of ≤1 mm	The leaves were not damaged, or only had needle-like (≤1 mm) wormholes on the leaves	Leaves were not damaged or only a few pinholes on the leaves were damaged
2	Only a few individual leaves have 3 to 6 wormholes with a diameter of no more than 1 mm	Only a few insect holes of the size of bullet holes (≤5 mm) are found on individual leaves	There are pinhole damage symptoms and circular point-like semi-transparent film window holes have appeared
3	There are more than 7 wormholes with a diameter of ≤1 mm on a few leaves	A few leaves have wormholes the size of bullet holes (≤5 mm)	There are a few small (5 to 10 cm) slender semi-transparent film window holes on the leaves
4	There are 1 to 2 wormholes with a diameter of no more than 2 mm on individual leaves	Notches (≤10 mm) on individual leaves	There are a few large (10–30 cm) membrane window holes on the leaves
5	A few leaves have 3 to 6 wormholes with a diameter of no more than 2 mm	A few leaves have notches (≤10 mm)	Large film window holes appear in blocks on the leaves, and the holes are less than one-third of the leaf area
6	There are more than 7 wormholes with a diameter of no more than 2 mm on some leaves	There are notches (≤10 mm) on some leaves	The area of window holes or openings on the leaves is large and accounts for about one-third of the total leaf area
7	A few leaves have 1 to 2 wormholes with a diameter greater than 2 mm	Some leaves were partially eaten, and a few leaves had large notches (≤10 mm)	The area of window holes or openings on the leaves is large and accounts for about half of the total leaf area
8	There are 3 to 6 wormholes with a diameter greater than 2 mm on some leaves	A few leaves were eaten, and some leaves had large notches (≤10 mm).	The leaves survived and the entire leaf contained large window holes or holes that were damaged
9	Most of the leaves have more than 7 wormholes with a diameter greater than 2 mm	Most of the leaves are eaten	The leaves were completely destroyed and almost all of the leaf surfaces were damaged

**Table 3:** Evaluation criteria for the resistance of corn leaves to *O. furnacalis*, *M. separata* and *S. frugiperda.*

Pest Damage Level	Average Leaf-Eating Grades of *O. furnacalis* During V5 Stage	Average Leaf-Eating Grades of *M. separata* and *S. frugiperda* During V5 Stage	Resistance
1	1.0–2.9	1.0–2.0	High resistance (HR)
3	3.0–4.9	2.1–4.0	Resistance^®^
5	5.0–6.9	4.1–6.0	Medium resistance (MR)
7	7.0–8.9	6.1–8.0	Sense (S)
9	9.0	8.1–9.0	High sense (HS)

**Table 4:** Grading criteria for the degree of damage caused by *O. furnacalis*, *H. armigera* and *S. frugiperda* during the corn ear stage.

Damage Level of Female Ear	Description of Symptoms of *O. furnacalis*	Description of Symptoms of *H. armigera*	Description of Symptoms of *S. frugiperda*
0	-	The ear is not harmed	-
1	The ear is not harmed	Only the silk was damaged	There is no breakage or only slight breakage in the silk, and the tips of the ear are not damaged
2	The damage rate of silk is less than 50%	The ear tip was damaged 1 cm	-
3	Damage rate of most filaments ≥50%; live larvae have been observed, and no more than second-instar	The ear tip was damaged 2 cm	The tip of the ear was slightly damaged, and the affected area was within 5% of the ear area
4	The ear tip was damaged ≤1 cm, or live larvae have been observed, and no more than third -instar	The ear tip was damaged 3 cm	-
5	The ear tip was damaged ≤2 cm, or live larvae have been observed, and no more than fourth -instar, the tunnel length less than 2 cm	The ear tip was damaged 4 cm	The affected area of ear accounted for 6–10% of ear area
6	The ear tip was damaged ≤3 cm, or live larvae have been observed, and more than fourth -instar, the tunnel length less than 4 cm	The ear tip was damaged 5 cm	-
7	The ear tip was damaged ≤4 cm, the tunnel length less than 6 cm	The ear tip was damaged 6 cm	The affected area of ear accounted for 11%~30% of ear area
8	The ear tip was damaged ≤5 cm, the tunnel length less than 8 cm	The ear tip was damaged 7 cm	-
9	The ear tip was damaged >5 cm, the tunnel length more than 8 cm	The ear tip was damaged 8 cm	The affected area of ear accounted for more than 30% of ear area

**Table 5:** Evaluation criteria for resistance of corn ears to *O. furnacalis*, *H. armigera* and *S. frugiperda*.

Mean Value of Damage to Ear	Resistance Type
1.0–2.0	High resistance (HR)
2.1–3.0	Resistance (R)
3.1–5.0	Medium resistance (MR)
5.1–7.0	Sense (S)
≥7.1	High sense (HS)

**Table 6:** Significant differences in corrected mortality were analyzed using Kruskal–Wallis nonparametric test.

Time	*O. furnacalis*			*M. separata*			*H. armigera*			*S. frugiperda*		
	df	*H*	*P*	df	*H*	*P*	df	*H*	*P*	df	*H*	*P*
DAY1	6	19.636	0.003	2	2.497	0.287	4	11.046	0.026	6	18.487	0.005
DAY2	6	19.274	0.004	2	6.058	0.048	4	11.533	0.021	6	18.628	0.005
DAY3	6	18.312	0.005	2	0	1	4	12.722	0.013	6	19.422	0.004
DAY4	6	17.176	0.009	2	0	1	4	12.470	0.014	6	18.911	0.004
DAY5	6	19.860	0.003	2	0	1	4	12.771	0.012	6	19.253	0.004

## Data Availability

Data are contained within the article.
